# Synaptic Effects of Palmitoylethanolamide in Neurodegenerative Disorders

**DOI:** 10.3390/biom12081161

**Published:** 2022-08-22

**Authors:** Martina Assogna, Francesco Di Lorenzo, Alessandro Martorana, Giacomo Koch

**Affiliations:** 1 Department of Clinical and Behavioural Neurology, Santa Lucia Foundation IRCCS, 00179 Rome, Italy; 2 Memory Clinic, Department of Systems Medicine, University of Tor Vergata, 00133 Rome, Italy; 3 Department of Neuroscience and Rehabilitation, University of Ferrara, 44121 Ferrara, Italy

**Keywords:** PEA, neuroinflammation, neurodegeneration, synaptic plasticity, frontotemporal dementia, Alzheimer’s disease, amyotrophic lateral sclerosis, transcranial magnetic stimulation, endocannabinoids

## Abstract

Increasing evidence strongly supports the key role of neuroinflammation in the pathophysiology of neurodegenerative diseases, such as Alzheimer’s disease, frontotemporal dementia, and amyotrophic lateral sclerosis. Neuroinflammation may alter synaptic transmission contributing to the progression of neurodegeneration, as largely documented in animal models and in patients’ studies. In the last few years, palmitoylethanolamide (PEA), an endogenous lipid mediator, and its new composite, which is a formulation constituted of PEA and the well-recognized antioxidant flavonoid luteolin (Lut) subjected to an ultra-micronization process (co-ultraPEALut), has been identified as a potential therapeutic agent in different disorders by exerting potential beneficial effects on neurodegeneration and neuroinflammation by modulating synaptic transmission. In this review, we will show the potential therapeutic effects of PEA in animal models and in patients affected by neurodegenerative disorders.

## 1. Introduction

As life expectancy is continuously rising, the global economic effect of neurodegenerative disorders, such as Alzheimer’s disease (AD), amyotrophic lateral sclerosis (ALS) and frontotemporal dementia (FTD) is increasing significantly [1]. The pathogenic mechanisms driving neurodegenerative illnesses, however, are still unknown. Several factors are involved, including genetic, environmental, and endogenous influences. Pathophysiological causes include abnormal protein dynamics, oxidative stress with reactive oxygen species, mitochondrial dysfunction, DNA damage, synaptic deficits, and neuroinflammatory processes [2].

Neuroinflammation is a complex process mediated by cytokines, which are primarily generated by microglia and astrocytes and whose activation can be harmful or protective to neurons. When implicated in the induction and control of neuronal development, cell survival, and synaptic plasticity pathways, beneficial pro-inflammatory cytokines are protective. Prolonged and abnormal pro-inflammatory signaling, however, is responsible for tissue neurodegeneration [3].

From a neuropathological point of view, neurodegenerative diseases are characterized by the deposition of misfolded proteins, such as amyloid beta (Aβ) and tau aggregates for AD and TAR DNA-binding protein 43 (TDP-43) in ALS and FTD. The progressive accumulation of these proteins triggers various pathological phenomena that contribute to the pathophysiological cascade of events that lead to the onset of clinical symptoms. Especially the impairment of the synaptic efficacy and the trigger and sustenance of neuroinflammation processes are increasingly being studied in neurodegenerative disorders as it has been shown their pivotal role in the progression of neurodegeneration and their potential modulation as therapeutic target in neurodegenerative diseases.

The aim of this review is to give insights into the interplay between synaptic machinery and neuroinflammation processes in neurodegenerative disorders and to clarify how palmitoylethanolamide (PEA)—an endogenous lipid mediator with high affinity for endocannabinoid receptor—and its new composite—which is a formulation constituted of PEA and the well-recognized antioxidant flavonoid luteolin (Lut) subjected to an ultra-micronization process (co-ultraPEAlut)—might be able to modulate this relationship in animal models and in patients.

### 1.1. Synaptic Impairment and Neuroinflammation in Neurodegenerative Disorders

#### 1.1.1. Alzheimer’s Disease

##### Synaptic Impairment

AD is macroscopically characterized by brain atrophy while microscopic hallmarks are the deposition of amyloid plaques and neurofibrillary tangles. Recently, with the introduction of biomarkers able to reflect in vivo the neuropathological alterations occurring in the disease, substantial modifications have been posed to AD definition; however, the clinical course of the disease remains unpredictable due to the scarce comprehension of pathophysiological mechanisms.

At this regard, there is strong evidence that synaptic density loss occurs before neuronal death, implying that impaired synaptic plasticity processes play a major role in AD etiology [4,5]. The loss of synaptic density has been reported to have the strongest statistical link with the degree of cognitive impairment in AD, rather than Aβ plaques, tangle formation, or neuronal death [6].

As a result, synaptic transmission impairment caused by toxic oligomeric species [7] can predict disease severity more accurately than gross neuronal death—a later occurrence—establishing synaptic dysfunction as a fundamental driver of AD-related cognitive decline rather than a byproduct [8]. Indeed, experimental studies in AD animal models have shown that Aβ peptides and tau proteins interact with physiological mechanisms of neuronal synaptic plasticity [9,10].

Moreover, N-methyl-d-aspartate receptor (NMDAr) mediated glutamatergic neurotransmission is crucial for synaptic plasticity and survival of neurons. Nevertheless, excessive NMDAr activity, mediated by excessive Ca^2+^ influx, may result in excitotoxicity and promotes cell death underlying a potential mechanism of neurodegeneration [11]. In humans, neurophysiological techniques, such as transcranial magnetic stimulation (TMS), can help in differentiating different neurodegenerative diseases [12] and forecast AD disease progression by estimating cortical functioning at a specific time [13].

TMS can be used to examine cortical plasticity mechanisms, such as long-term potentiation (LTP), one of the most important neurophysiological correlates for learning and memory [14]. We previously demonstrated that AD patients had a consistent deficit of LTP-like cortical plasticity in motor function [15,16] and the cerebellar cortex [17], with a sparing of mechanisms of long-term depression (LTD), evident also in early mild cognitive impairment (MCI) patients [18].

Moreover, in AD animal models, the synaptic dysfunction has been linked to a disorder of high-frequency neuronal oscillatory activity, in particular in the gamma range (40 Hz) [19,20]. Accordingly, in a recent work, TMS combined with EEG (TMS-EEG) recordings have shown that AD patients had more prominent decrease in gamma activity in the prefrontal cortex with a stronger impairment of LTP-like plasticity mechanisms and more prominent cognitive decline [21]. Interestingly, the optogenetic entrainment of fast-spiking parvalbumin-positive interneurons of AD animal model at gamma frequencies was able to reduce the total amyloid levels, probably acting on both neurons and microglia [22].

Similarly, intranasal administration of pro-resolving lipid mediator in a mouse model of AD was able to improve memory dysfunction and restore gamma oscillation impairment, accompanied by a modulation of microglial activation [23].

##### Neuroinflammation in AD

Microglial cells are a primary target of neurodegenerative disease research because they play a vital part in the inflammatory process of the central nervous system. Depending on the specific stimulus the microglia have been exposed to, it could maintain a balance between a pro-inflammatory status (M1 phenotype), characterized by the synthesis of inflammatory cytokines, such as interleukin 1 (IL-1), interleukin 6 (IL-6), and tumor necrosis factor (TNF), and the synthesis and release of anti-inflammatory cytokines (IL-4, IL-8, and IL-10) and neurotrophic factors (M2 phenotype) [24]. Thus, the complex involvement of inflammatory cytokines in both neurodegeneration and neuroprotection is far from complete in such a complex environment. Amyloid peptides, comprising both oligomeric and senile plaque forms, are thought to be the key inflammatory trigger in AD. A prolonged pro-inflammatory signaling caused by amyloid mis-metabolism, in particular, can result in an overproduction of pro-inflammatory cytokines involved in neurodegenerative pathways signaling [3]. While there is evidence that persistent neuroinflammation causes an increase in amyloid synthesis [25], a clear relationship between tau pathology and neuroinflammation is still unclear. We recently showed that human astrocytes cultures incubated with cerebrospinal fluid (CSF) samples from AD patients were vulnerable in terms of increased apoptosis only in the presence of high levels of tau protein and APOE4 genotype [26].

**Figure 1:**
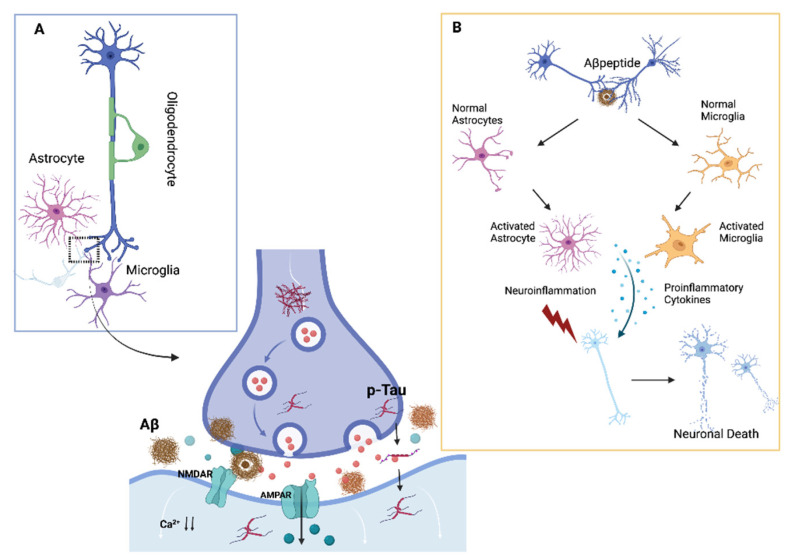
**Neuroinflammation and synaptic impairment in AD:** Panel (**A**) displays the interplay among the neuron and the glial cells involved in AD pathology, such as astrocyte, microglia, and oligodendrocyte. In the dotted square, the particular of synaptic transmission impairment induced by Aβ and p-Tau activation; panel (**B**) shows the probable mechanisms of action of the activated microglia and astrocyte induced by amyloid beta deposition and the pro-inflammatory cytokines cascade in the pathophysiology of AD. AD: Alzheimer’s disease; Aβ: amyloid beta; p-Tau: phosphorylated tau.

As a result of these observations, we hypothesized that tau proteins play a substantial role in astrocyte degradation and a proinflammatory role in APOE4 patients [27]. Surprisingly, APOE4 carriers have been found to have an imbalanced flipping of the microglial phenotype M1–M2 [28]. Furthermore, microglial apolipoprotein E (ApoE) regulates microglial homeostatic gene expression downstream, resulting in a neurodegenerative phenotypic switch that could exacerbate AD pathogenesis [29]. Consistent with this framework, we showed that during early phases of AD, in APOE4 carriers, amyloid pathology likely induces a specific cytokines pattern synthesis associated to cognitive preservation [30] (Figure 1).

#### 1.1.2. Frontotemporal Lobar Degeneration

##### Synaptic Impairment in FTLD

Similar to AD, synaptic disruption appears to precede neuronal death also in frontotemporal lobar degeneration (FTLD). Indeed, FTLD abnormal brain connectivity (connectopathy) or synaptopathy has been described [31,32]. In the affected cortex, extensive synaptic loss and a reduction in the number of spines have been shown post-mortem [33,34]. In some [35], but not all, studies [36], a significant decrease in synaptic density evaluated with synaptophysin in the superficial layers of the prefrontal cortex of FTLD individuals compared to normal controls was described. In Pick’s illness, synaptophysin immunoreactivity was likewise diminished in the hippocampus dentate gyrus’ outer molecular layer [37].

Recently, in a sample of behavioral variant of FTD (bvFTD), synaptic loss was measured in vivo with synaptic vesicle glycoprotein 2A (SV2A)-PET, a metabolic marker of synaptopathy, in the anterior parahippocampal gyrus of a sample of bvFTD patients [38].

Interestingly, in a TMS study, altered mechanisms of plasticity were observed also in pre-symptomatic FTD carriers of progranulin (GRN) and C9orf72 genes mutation in comparison to age-matched healthy controls, reinforcing the notion that alteration of synaptic machinery begins years before the onset of clinical symptoms [39].

##### Neuroinflammation in FTLD

Neuroinflammation and immune-mediated processes have been identified as key contributors to the degenerative process of FTD [40,41,42]. Neuroinflammation is a hotly contested topic [43], whether it is a main or secondary event in the neurodegeneration associated with FTD or has an overall helpful or negative effect. Alternatively, the initial pathological insult (that is, aggregation and/or accumulation of amyloid-, tau-, or TDP43) induces an ongoing cytotoxic response that results in secondary chronic neuroinflammation and altered neuronal function in brain regions specific to the disease phenotype [44,45]. The buildup of aberrant conformations of tau or TDP43 signals generated by injured neurons [46] or deregulation of the systems for clearing misfolded or damaged neuronal proteins are likely to stimulate immune activation in FTD. These mechanisms eventually result in neurodegeneration [47,48].

Depending on the stage and severity of the disease, many immunological mechanisms, both innate and adaptive, are likely to be engaged. As a result, looking into neuroinflammatory and immune-mediated pathways for diagnostic biomarkers, innovative therapy targets, and disease-modifying medicines for FTD is a potential line of research.

#### 1.1.3. Amyotrophic Lateral Sclerosis

##### Synaptic Impairment in ALS

Amyotrophic lateral sclerosis (ALS) is a neurodegenerative illness characterized by motor neurons loss (MNs). Misfolded proteins, glutamate excitotoxicity, mitochondrial dysfunction at distal axon terminals, and alterations in the neuronal cytoskeleton are all pathogenic characteristics of ALS. C9orf72 gene-mediated pathogenesis is aided by synergies between the loss in C9orf72 functions and the gain in function caused by toxic consequences of repeat expansions [49]. Neuropathological hallmarks of ALS are dendritic and synaptic degeneration in the cortex and corticospinal (CS) motor neurons [50].

Braak and colleagues classified ALS as a disease of big axon neurons with different stages of trans-synaptic dissemination based on the cortical synaptic degeneration hallmark [51]. According to this classification, the disease begins in corticospinal motor neurons (CSMNs), develops to MNs, and then to extra-motor regions [51,52]. Cortical hyperexcitability is an early clinical characteristic of ALS, and this pathological insight is consistent with it [53]. Furthermore, the trans-synaptic spread theory implies a prion-like mechanism for spreading misfolded protein aggregates to distant populations of neurons [51]. This supports the theory that ALS is caused by a synaptopathy [54].

Synaptopathy is a general term for disorders characterized by synaptic dysfunction, independent of the underlying causes [55]. Synaptopathy, in its broadest sense, refers to a collection of symptoms that, over time, contribute to synaptic failure. Changes in Ca^2+^ levels at synapses, glutamate excitotoxicity, structural changes in pre- and postsynaptic anchoring proteins, altered synaptic structure and function, which is frequently associated with dendritic spine loss, dysfunctional neurotransmitter release, impaired maintenance and regeneration of axons by Schwann cells, and cognitive deficits are among these characteristics [54,55]. Neuronal loss, mitochondrial dysfunction, accumulation of misfolded proteins associated with faulty proteostasis, and dysfunctional neuromuscular junctions are all symptoms of synaptopathies [55,56].

Several studies have shown aberrant synapses’ shape and function in ALS, with the nature of the abnormalities varying depending on the disease’s progression [54,57]. In addition, neuronal loss, alterations in dendritic spine density, and morphology in excitatory neurotransmission locations, particularly in pyramidal cells, such as CSMNs, have been documented in ALS post-mortem samples [54,57]. This trait has also been seen in many ALS animal models [58]. We will examine how alterations in multiple pathways can contribute to synaptic dysfunction in the C9orf72-ALS pathogenesis in this review.

##### Neuroinflammation ALS

Neuroinflammation is a pathogenic process defined by the invasion of activated microglia and astrocytes in ALS patients with and without genetic abnormalities. In post-mortem tissues of ALS patients, activated microglia and astrocytes that produce pro-inflammatory cytokines are increased [59,60,61]. In living ALS patients, a PET investigation revealed increases in activated microglia [11C-(R)PK11195 PET] and astrocytes (11C-DED PET) [62,63]. Furthermore, sporadic ALS patients with varied degrees of disease severity have considerably greater CSF soluble triggering receptor expressed on myeloid cells 2 (sTREM2) levels than controls [64]. CSF sTREM2 levels are highest in early-stage ALS, and higher levels of CSF sTREM2 are associated with slower disease development in late-stage ALS. High levels of sTREM2 in the CSF for a long time could indicate a neuroprotective phenotype [64].

## 2. PEA and PEA Combined with Luteolin Mechanisms of Action

### 2.1. PEA Synaptic Mechanisms of Action

PEA is an endogenous lipid mediator, which belongs to the class of acylethanolamides (AEs), produced “on demand” from phospholipids membranes with a well-recognized anti-inflammatory, analgesic, and neuroprotective function in various conditions in both central and peripheral nervous system [65,66,67,68]. The biological effect of PEA has been extensively investigated by preclinical studies, and it seems to be mediated by the direct activation of the orphan GPCR 55 receptor (GPR55), while the affinity for type-1 and type-2 cannabinoid receptors (CB1 and CB2) and for the transient receptor potential vanilloid 1 (TRPV1) channels is lower. Moreover, PEA plays a central role in the modulation of pain and inflammation pathways by the activation of peroxisome proliferator-activated receptor-alpha receptor (PPAR-α) [69,70]. In particular, the activation of PPAR-α induced by PEA, through the interaction with transcriptions factors is involved in the reduction in NF-κB activation and pro-inflammatory enzyme synthesis, thus promoting anti-inflammatory and analgesic effects [71,72]. Moreover, the protective effects of PEA in neurodegeneration and neuroinflammation preclinical models of different pathologies are reversed by the pharmacological modulation of PPAR-α with antagonists or its genetic silencing [73,74,75,76]. GPR55 receptors are broadly expressed in several brain areas, and they were identified as cannabinoid receptors with a different signaling pathway from CB1 and CB2 receptors [77]. The PEA-induced anti-inflammatory effects seems in part to be mediated by the activation of GPR55 in an experimental murine model of colitis [78], in a murine model of Parkinson’s disease [79], and in chronic arterial inflammation [80]. While the immunomodulatory function of GPR55 is widely recognized, their effect on neuronal cells and their localization is still elusive [81]. A recent work from Musella et al., [82] investigated the involvement of PEA in both excitatory and inhibitory transmission in the striatum of a rodent model. The authors found for the first time that PEA can enhance GABA transmission and modulate the synthesis of 2-Arachidonoylglycerol (2-AG), which acts as inhibiting in a retrograde manner at a presynaptic site the CB1R and GABA release. Furthermore, PEA can modulate and enhance indirectly the levels of other endocannabinoids, through the so-called entourage effect [65,67]. Despite the low affinity for CB1 and CB2 receptors exerted by PEA, they can be activated indirectly in different ways. Indeed, PEA can reduce the degradation of anandamide (AEA), acting on the fatty acid amide hydrolase (FAAH), the enzyme responsible for endocannabinoids degradation, leading to an increase in the cannabinoid receptor mediated transmission. More recently, PEA exposure was found to induce changes in microglia morphology and activation through the PPAR-α activation, including increased migration and phagocytosis due to a reactive microglial phenotypes mediated by its indirect regulation of CBR2 receptors [83]. In addition, in a recent study from D’Aloia et al., the treatment with PEA inhibited the M1 microglial polarization induced by lipopolysaccharide (LPS), while the anti-inflammatory markers in microglial cells were upregulated, highlighting a possible role for PEA in inducing the anti-inflammatory M2a phenotype and as a potential therapeutic tool in neurodegenerative disorders with chronic microglial hyperactivity [84]. Finally, PEA can also indirectly modulate other endocannabinoids targets, such us the TRPV1 channel [85,86]. Interestingly, it has been recently reported that TRPV1 activation in animal models of Parkinson’s and AD can exert neuroprotective effects [87,88,89,90]. Accordingly, the role of the TRVP1 activation by the endocannabinoid anandamide was found to be effective in reversing memory deficits and hippocampal function from Aβ-induced cytotoxicity, in rodents, in particular through the effect of rescues of gamma oscillations [90], which an alteration is emerging as a key mechanism for the pathophysiology of AD [91]. The administration of the TRPV1-receptor agonist capsaicin was able to restore hippocampal damage and gamma oscillations by reversing both the desynchronization of action potential firing in CA3 pyramidal cells and the shift in excitatory/inhibitory current balance, thus suggesting the pathway as a possible therapeutic target for AD [90].

Noteworthy, PEA seems to exert neuroprotective effects also through the modulation of the glutamatergic transmission and synaptic plasticity. Indeed, Lin and colleagues showed that in rat cerebrocortical nerve terminals, PEA could act on glutamate pathways exerting a presynaptic inhibition of glutamate, likely through a reduction in the Ca^2^⁺ influx, which might be linked to the activation of presynaptic cannabinoid CB1 receptors [92]; the inhibition of aberrant glutamatergic activity might thus result in an anti-excitotoxic effect, which could counteract the neurodegenerative process.

All these findings strongly support that PEA could be considered as a promising molecule to counteract neuroinflammation and neurodegeneration through its multiple synaptic targets.

### 2.2. PEA Combined with Luteolin Effects

Several studies have been shown a role of flavonoids, in particular luteolin, in displaying many neuroprotective and anti-inflammatory properties in chronic conditions and neurodegenerative disorders [93]. Moreover, luteolin has been shown to exert anti-oxidant activity and to improve glucose metabolism by potentiating insulin sensitivity and modulating Aβ deposition by the activation of the gut-microbiota-liver-brain axis in AD models [94]. The combination of the pharmacodynamic properties of PEA and those of luteolin was found to be more effective in counteracting both inflammation and oxidative stress. Accordingly, many studies have shown that using PEALut can provide better effects by stimulating both hippocampal neurogenesis and dendritic spine maturation [95,96] and that the two molecules potentiate their synergic effect when simultaneously submitted to the micronization process [67]. Additionally, the association with flavones seems to stabilize the two molecules and enhance their pharmacological activities [97], even in experimental models of AD and other neurodegenerative disorders [76,98], mainly through the modulation of the neuroinflammatory and apoptotic pathways, the cytokines release, the activation of astrocytes and microglia, and the ability to modulate the autophagic process [95,99].

## 3. Synaptic Effects of PEA and PEALut in Neurodegenerative Disorders

In the last few years, PEA has been identified as a potential therapeutic agent in different neurodegenerative disorders [100,101]. Based on several preclinical and clinical studies, PEA can exert potential beneficial effects on neurodegeneration and neuroinflammation by modulating synaptic transmission.

### 3.1. Preclinical Models

The first in vitro study of the mechanism of PEA in AD model from Scuderi et al. in 2011 demonstrated the ability of PEA to reduce Aβ-induced astrocyte activation and proinflammatory molecules and cytokine release in primary rat astrocytes through a PPAR-α-dependent mechanism [72]. In line with the same observations, a later study of the same authors showed that PEA reduced reactive gliosis and attenuated neuronal damage in rat models of Aβ-induced neurotoxicity, with a mechanism strictly depending on PPAR-α activation [102,103].

Accordingly, the neuroprotective effect of PEA was demonstrated in mice injected with amyloid-β 25–35 (Aβ 25–35) peptide intracerebroventricularly, evaluated for learning and memory deficits [73]. Surprisingly, PEA was able to reduce or prevent, in a dose-dependent manner, the Aβ induced behavioral deficits, while it failed to rescue memory impairment in peroxisome proliferator-activated receptor-α (PPAR-α) null mice, thus further highlighting the importance of PPAR-*α* modulation for neuroprotection and PEA efficacy against amyloid neuronal damage [73].

A following study from Scuderi et al. in 2014 investigated the systemic administration of PEA in murine models given an injection of beta-amyloid 1–42 (Aβ 1–42) in the hippocampal cortex, to further elucidate its therapeutic potential and the mechanisms underlying the behavioral effects. The authors reported significant modifications in biomarkers related to astrogliosis and amyloidogenesis, finding new evidence that PEA can restore behavioral deficits and impaired molecular pathways similar to early traits of AD by activating PPAR-α [104].

The anti-inflammatory properties of the co-ultraPEALut, a composite of PEA and the antioxidant flavonoid luteolin, has been investigated in organotypic model of AD by incubating with Aβ1-42 peptide differentiated human neuroblastoma cells and hippocampal slice cultures [75]. The authors showed that the compound exerted a protective effect on glial cells by reducing significantly apoptosis and glial fibrillary acidic protein expression and restoring neuronal nitric oxide synthase and brain-derived neurotrophic factor [75].

Accordingly, the incubation with co-ultraPEALut significantly reduced the TNF-α-induced serum amyloid A (SAA) mRNA expression in oligodendrocyte precursor cells, which is relevant since SAA has been demonstrated to localize immunohistochemically with aβ deposits in AD brain [105].

Moreover, co-ultra PEALut was able to counteract the Aβ1–42-mediated inflammation and astrocyte reactivity in an in vitro model of AD and to restore oligodendrocytes homeostasis through a mechanism that could involve PPAR-α activation [106]. Interestingly, PEA effects in neuroinflammation and AD angiogenesis were demonstrated in Aβ-treated C6 rat astroglioma cells and human umbilical vein endothelial cells (HUVEC) [107]. As expected, PEA was able to induce a dose-dependent reduction in pro-inflammatory and pro-angiogenic biomarker in the cells stimulated with Aβ, and the effect was blocked in the model by the treatment with the PPAR-α antagonist GW6471, further suggesting the mechanism on astroglial cells is proliferator-activated receptor alpha-dependent [107].

Similarly, PEA was tested for its neuroprotective effects against Aβ-induced toxicity on cell vitality and glutamatergic transmission in AD mice, particularly in primary cultures of cortical neurons and astrocytes from mice with the triple-transgenic AD model (3×Tg-AD) and the wild type mice [108]. As expected, PEA reversed the effects of the Aβ1-42 fragment on glutamatergic transmission and cell viability in cultured neurons and astrocytes isolated from wild type mice. On the contrary, PEA did not prevent the formation of Aβ-plaques and neurofibrillary tangles or early synaptic dysfunction or cognitive decline in 3×Tg-AD mice, thus suggesting a possible efficacy only in early AD [108].

Another study investigated the reactive astrogliosis process in 3×Tg-AD mice treated with PEA; the authors found that the astrocytes’ reactive state and neurons’ viability were improved by PEA revealing its beneficial neuro-supportive function [109].

Interestingly, PEA also has been shown to exert immunomodulatory, analgesic, and neuroprotective effects and restore cognitive dysfunction in different chronic pain conditions by restoring glutamatergic synapses’ functioning deficits [110,111,112].

In particular, the involvement of metabotropic glutamate receptor (mGluR) 5 and 8 in ultra-micronized (um-PEA) effects on cognition and long-term potentiation (LTP) mechanisms was investigated in the entorhinal cortex (LEC)-dentate gyrus (DG) pathway in mouse models of spare nerve injury (SNI) [113].

The chronic treatment with this compound rescued discriminative memory and LTP deficits at the LEC-DG pathway in SNI mice. The authors, based on the physiological role exerted from glutamate in memory formation processes in the hippocampus hypothesized that the modulation of glutamatergic activity might be at the base of the PEA-induced restoration of LTP and cognitive behavior in SNI models of chronic pain, thus reducing glutamate excitotoxicity [111]. In particular, the administration of mGluR5 antagonist facilitated memory and plasticity mechanisms while the mGluR8 blockage prevented the protective action of the PEA on LTP, thus displaying different roles but both necessary to mediate the efficacy of PEA in neuropathic pain [111].

In a recent work from Beggiato et al. from 2020, the authors investigated the effects of ultra-micronized PEA (um-PEA) treatment in 3×Tg-AD mice, and they found that it was able to reduce the typical increase in hippocampal glutamate levels observed in the AD mouse model [114].

Accordingly, chronic treatment with PEA reversed memory deficit and LTP impairment in SNI wild type mouse models, but not in PPARα null mice, and restored glutamatergic transmission deficits, the loss in synaptic density, and the expression of phosphorylated GluR1 subunits, as well as an increase in neuroblasts [111]. Moreover, the increase in synaptogenesis induced by PEA in SNI mice was correlated to the improvement in episodic memory and LTP. Taken together, these results open new perspectives for the use of PEA for the translation of the same result in AD pathology for which an impaired or a same synaptic transmission deficit and impairment of LTP mechanisms have been demonstrated [12].

The therapeutic potential of three-months subcutaneously administration of ultra-micronized PEA (um-PEA) was investigated in 3×Tg-AD mice, and mitochondrial bioenergetics alterations, which can lead to glutamatergic neurotransmission alterations and excitotoxicity, were evaluated in the frontal cortex (FC) and hippocampus (HIPP), with the results showing that um-PEA was able to counteract mitochondrial dysfunctions and rescue brain energy metabolism in the FC but not in the HIPP [115].

Local synaptic effects of PEA on GPR 55 transmission in ventral-hippocampus (vHipp), a key region to memory function, and the consequent modulation of mesolimbic activity were investigated in in vivo rat brains from Kramar et al., 2017 [116].

As hypothesized, vHipp GPR55 activation was found to increase glutamatergic levels in the hippocampus, potentiating excitatory transmission from the vHipp to the mesolimbic cortex, including the ventral tegmental area (VTA).

According to the result of this study, PEA administration was found to increase VTA dopaminergic frequency and bursting rates through a local NMDA-receptor dependent mechanism [116].

Finally, the pharmacological properties of co-ultra PEALut were investigated in an in vivo mouse model of prodromal AD. In Aβ infused rats, the early administration of PEA reduced the astrogliosis and microgliosis and prevent the over-expression of pro-inflammatory cytokine genes and the reduction in mRNA levels BDNF and GDNF, which are fundamental neurotrophins regulating synaptic plasticity mechanisms and neuronal growth and branching [117].

### 3.2. Clinical Studies

Thus far, several studies reported the protective effects of PEA in neuropathic pain and peripheral conditions sustained by neuroinflammation, while there are few works aimed at investigating the efficacy of PEA administration in neurodegenerative disorders in humans.

The first case report on um-PEA oral administration in a patient with ALS showed an improvement of the clinical picture, as measured by electromyographic analysis and respiratory capacity, due probably to the ability to modulate neuroinflammation [118]. According to this previous finding, a larger study on ALS tested the clinical and molecular effects of 600 mg um-PEA administration (in 28 patients) twice daily as an addition to standard therapy alone (50 mg riluzole, 36 patients) for six months [119].

Surprisingly, the trial showed that PEA-treated ALS patients had a slower decline in respiratory function as measured by a lower decrease in their forced vital capacity (FVC) and later need for a tracheotomy, compared to the untreated patients.

Moreover, the authors, by micro-transplanting human muscle membranes from muscle biopsies of ALS patients into Xenopus oocytes, showed that PEA can reduce the desensitization of AChRs-evoked currents in both ALS and non-ALS human samples after repetitive neurotransmitter application, which is selectively effective on human e-AChRs subtype, thus providing molecular basis for PEA efficacy on muscle excitability and evidences on acetylcholine modulation exerted by the compound [119].

Until now, there are no clinical data published on the possible beneficial effects of PEA in AD patients. Only one study investigated the efficacy of nine months PEALut high-dose administration in amnestic MCI in a patient [120].

The patient at baseline underwent a neuropsychological examination, which included attentive matrices, Babcock Story recall test, Mini-Mental State examination (MMSE), Montreal Cognitive Assessment, Rey Auditory-Verbal Learning Test, Trail Making Test, and verbal fluency tests as well as a perfusion single-photon emission computed tomography, which documented a significant hypoperfusion in the parietal, inferior-temporal, and temporo-occipital areas. At the nine-months follow-up, the neuropsychological evaluation was almost normal, and the SPECT hypometabolism was normalized [120], thus opening to the design of larger clinical trials in this population.

A recent study published by our group [121] investigated, for the first time, the cognitive and neurophysiological effects of four weeks of PEALut administration in seventeen patients with FTD. For the purpose of this study, patients underwent an extensive cognitive and behavioral assessment, which included neuropsychiatric inventory (NPI), MMSE, frontal assessment battery (FAB), screening for aphasia in neurodegeneration (SAND), FTLD-modified clinical dementia rating scale sum of boxes (FTLD-SOB.) To further investigate in vivo neurophysiological synaptic effects of the compound administration, we used paired-pulse and repetitive TMS protocols assessing LTP mechanisms and long-interval intracortical inhibition. Moreover, TMS-EEG recordings were collected to evaluate changes in frontal oscillatory activity.

**Figure 2:**
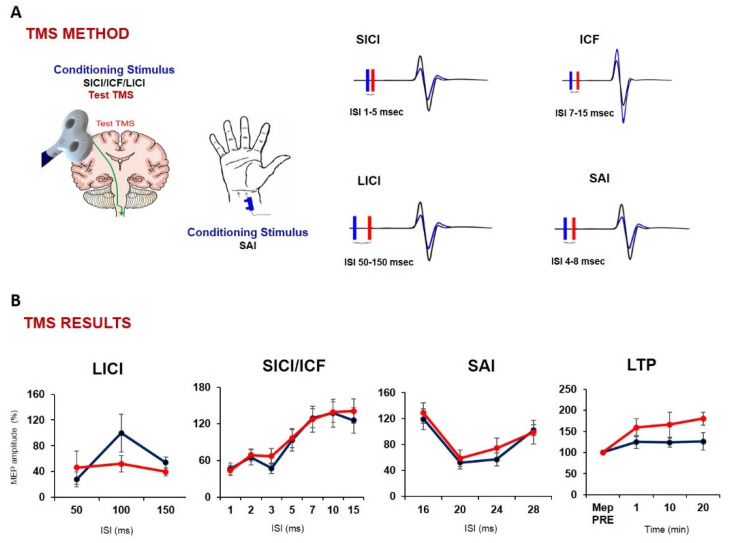
**Neurophysiological effects of PEALut in FTD patients: paired-pulse TMS results and iTBS after effects** (adapted from [121]): Panel (**A**) provides a schematic illustration of TMS protocols investigating synaptic transmission and cortical plasticity; panel (**B**) shows corticospinal measures. After one month of treatment with PEALut, we observed a significant restoration of LICI and LTP, suggesting a restoration of GABA(B) activity and cortical plasticity. No effects were found in protocols measuring cholinergic neurotransmission (SAI) and GABA(A) activity (SICI). (blue line represents the pre-treatment results, red line post-treatment). FTD: frontotemporal dementia; TMS: transcranial magnetic stimulation; PEALut: palmithoylethanolamide combined with luteolin; LICI: long-interval intracortical inhibition; LTP: long-term potentiation; SICI: short-interval intracortical inhibition; SAI: short-latency afferent inhibition; iTBS: intermittent theta burst stimulation.

**Figure 3:**
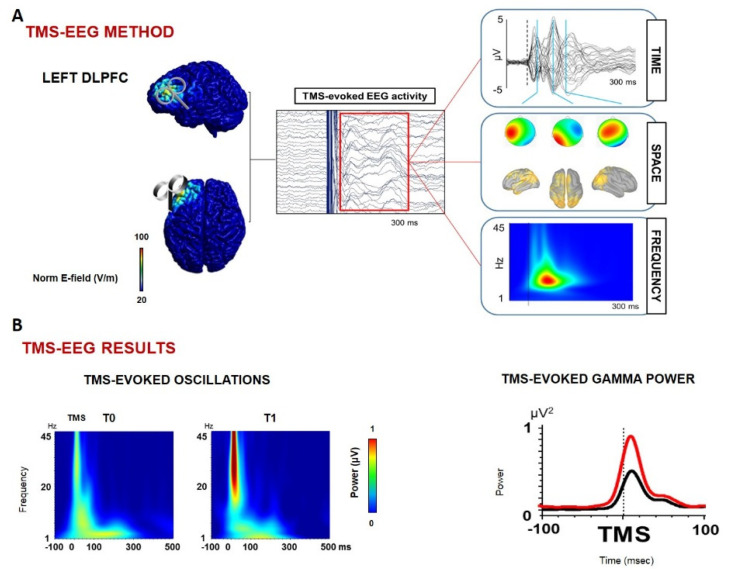
**Effects of PEALut on cortical oscillations in FTD patients** (adapted from [121]): Panel (**A**) provides a schematic illustration of TMS-EEG protocols investigating cortical reactivity, oscillatory activity, and connectivity on left DLPFC; panel (**B**) displays cortical measures results. After one month of treatment with PEALut, we observed a significant increase in high-frequency oscillations (black line represents the gamma power pre-treatment, red line post-treatment). FTD: frontotemporal dementia; TMS: transcranial magnetic stimulation; PEALut: palmithoylethanolamide combined with luteolin; DLPFC: dorsolateral prefrontal cortex.

Surprisingly, the results showed that PEALut can improve frontal lobe function and behavioral disturbances, mainly through the modulation of GABAergic activity and high-frequency cortical oscillatory activity, which is impaired in FTD patients [121] (Figure 2 and Figure 3).

## 4. Conclusions

So far, the pharmacological treatment for AD and other neurodegenerative dementias has been mostly based on symptomatic drugs enhancing cognition and reducing behavioral alterations (antipsychotic drugs, acetylcholinesterase inhibitors, and NMDA receptor antagonist) with contrasting results and no efficacy in modifying disease progression [122].

Passive immunotherapies based on the administration of exogenous antibodies targeting the two hallmarks in AD pathology, which are Aβ and tau protein, have given ambiguous results and minimal therapeutic benefit [123].

Based on this premise, a conceptual shift in the approach to neurodegenerative dementias treatment is needed urgently. In this context, new therapies focusing on synaptic dysfunction [124,125] and neurotransmitters deficits, as well as new recognized pathogenetic mechanisms, such as neuroinflammation, might be considered as a promising therapeutic agent to counteract neurodegeneration.

In this scenario and based on the results presented in this review, new compounds such as PEA, and new formulations, such as PEAlut, with its multiple pharmacological targets and mechanisms of action, fulfill the criteria for a potential promising key role in modulating neuroinflammation and synaptic neurotransmission, especially at early stages, thus modifying disease progression.

While preclinical studies data are promising, the lack of larger clinical trials is necessary to further elucidate the role of PEA and PEAlut in neurodegenerative diseases treatment.

## Data Availability

Not applicable.
